# Does Biosynthetic Silver Nanoparticles Are More Stable With Lower Toxicity than Their Synthetic Counterparts?

**Published:** 2019

**Authors:** Zohreh Rezvani Amin, Zahra Khashyarmanesh, Bibi Sedigheh Fazly Bazzaz, Zahra Sabeti Noghabi

**Affiliations:** a *Biotechnology Research Center, Pharmaceutical Technology Institute, Mashhad University of Medical Sciences, Mashhad, Iran. *; b *Department of Chemistry, Farhangian University, Tehran, Iran.*; c *Department of Medicinal Chemistry, School of Pharmacy, Mashhad University of Medical Sciences, Mashhad, Iran*.; d *Department of Pharmaceutical Control*, *School of Pharmacy, Mashhad University of Medical Sciences, Mashhad, Iran*.

**Keywords:** Biosynthesis, Purification, Silver nanoparticles, Staphylococcus aureus, Toxicity

## Abstract

Control of size and shape is a challenge in nanoparticle synthesis. Synthetic and biosynthetic (both extracellular and intracellular) methods are used to prepare silver nanoparticle (SNP). In this study, the behavior of three strains of *Staphylococcus aureus* (*S. aureus*) was investigated in the presence of silver nitrate intra- and extracellularly. *S. aureus* strains biosynthesized SNPs intracellularly, while in the method of the extracellular biosynthesis, none of the strains could produce the SNP under different conditions (dark, bright light, and the presence of nitrate ion). Intracellular SNPs were purified. The results of this study and previous results were used to compare different properties of the biosynthetic (intra- and extracellular) and synthetic SNPs in terms of shape, size, zeta potential, stability, and toxicity. The results confirmed lower toxicity of biosynthetic SNPs *in-vitro* assays, and their more stability with less aggregation compared to the synthetic ones. Also, the biosynthetic nanoparticles were found uniform and small. These nanoparticles may be useful for being employed as biosensors.

## Introduction

Nanoparticle synthesis often includes chemical or biological methods. Synthetic methods require chemical reducing agents. In biological method (biosynthetic method) the synthesis of nanoparticles is done by plant extracts or living organisms (bacteria, fungi, and actinomycetes) (1-2). Enzymes, which can cause enzymatic reduction of metal ions, were used as biological reagents. For example, the nitrate reductase enzyme is found to be responsible for the biosynthesis of nanoparticles such as silver nanoparticles (SNPs) (3). Biosynthesis that can be carried out at ambient temperature and pressure, also requires low concentration of reagents (4). These procedures are safer, nontoxic and low-cost techniques. Biosynthesis due to its environment friendly approach is important. Nanoparticles at biological synthesis are stabilized by available proteins and peptides in the environment. These are eco-friendly reagents (5). The biosynthesis of nanoparticles using microorganisms usually involves intracellular and extracellular techniques. In intracellular synthesis process, culture medium containing microorganism is mixed with metal salt solution, while in extracellular biosynthesis, the microorganism cells are separated from the culture medium and the cell-free extract is used for biosynthesis of nanoparticles. In the latter, metal salt solution is added to the supernatant. Generally, the biosynthetic steps of nanoparticles using microorganisms involve two steps including the biosynthesis of nanoparticles and recovery of the nanoparticles from microorganism components. Because of complex recovery of nanoparticles in intracellular biosynthesis, properties of obtained nanoparticles usually were not characterized completely. To solve this problem, the nanoparticles were biosynthesized using the supernatant of culture medium (extracellularly). However, in this nanoparticles the size distribution and/or shape could be diverse (6-10). The collective oscillation of conduction electrons in SNPs causes the surface plasmon resonance (SPR) phenomenon, and this produces a strong absorption in the visible region. SPR phenomenon is dependent on nanoparticle shape and size. This phenomenon is very important in the industry of biosensors (11). The purpose of this study was to evaluate different strains of *S. aureus* (ATCC 6538p, 29737, and 25923) for intra- and extracellular biosynthesis of SNPs. Biosynthetic nanoparticles were recovered, characterized, and tested for cytotoxicity and antibacterial properties. In addition, properties of biosynthetic nanoparticles including shape, size, zeta potential, stability, and cytotoxicity were compared with properties of those prepared chemically.

## Experimental

*Synthesis of SNPs using Sodium borohydride*

All glassware used was cleaned using mixture of HCl/HNO_3_ (3/1) and distilled water. 0.001M silver nitrate (5 mL) were added drop by drop (about 1 drop per second) to 30 mL of 0.002 M sodium borohydride solution that had been chilled in an ice bath. 

The solution was mixed vigorously on a magnetic stirrer. The solution turned to bright yellow when all of the silver nitrate had been added (12-13).

*Biosynthesis of SNPs using S. aureus (intracellularly) and purification steps*

Different strains of *S. aureus* (ATCC 6538p, 29737, and 25923) were separately grown in flasks containing 50 mL LB broth medium (US, Thermo Fisher Scientific) at pH 7.5, 35 °C, and 150 rpm in shaker incubator for 24 h (14). Next, 100 mL fresh LB broth and 10 mL aqueous solution of silver nitrate (2 mM) were added to 40 mL the culture. pH of the cultures were adjusted at 8 and the cultures were grown at 35 °C and 150 rpm for a further 24 h. After color change of the cultures (from white to brown and dark brown), they were incubated at room temperature for further 4 h. The content of each flask was centrifuged (15 min at 3634×g). The prepared pellets were washed with phosphate buffered saline (PBS) (1X). The purification steps of intracellular SNPs were performed (15) (see complete protocol in supplementary file). Gel electrophoresis was employed for characterization of SNPs. Gel electrophoresis separated SNPs by a 0.7% agarose gel (15 cm electrode spacing, ran for 10 min at 150 V) in Tris/borate/EDTA (TBE) buffer (0.5 X) at pH 9 (16). Two concentrations of SNPs (about 7 µL from 10 and 5 µg/µL) were added to the gel wells separately. The steps were performed in weak light conditions. 

*Biosynthesis of SNPs using S. aureus (extracellularly)*

The strains of *S. aureus* were grown (Luria-Bertani (LB) broth at pH 7.5 and 35 °C) under conditions of dark (17) and bright light (10) for 24 and 48 h. Also, some flasks with the same content plus 10 mmol KNO_3_ were treated with *S. aureus*. The cultures were centrifuged at 5232×g and their supernatants were used for biosynthesis of SNPs. Silver nitrate solution (10^−3^ M) was added to the supernatants (1%, v/v) and the reaction mixtures was allowed to stand at 25 °C for 24 h. 

*Characterization of SNPs*

The biosynthesis of SNPs was confirmed by color change of reaction mixture and UV-Visible spectroscopy analysis. Absorbance peak between 370-500 nm in UV-Vis spectroscopy indicates presence of SNPs (18). Measurement of the UV-Vis spectrum was performed by Shimadzu -1650 PC UV-Vis spectrophotometer operated at a resolution of 1 nm. The X-ray diﬀraction (XRD) technique was performed on samples (Unisantis, XMD-300, X-ray Diffractometer). All diffraction patterns were obtained in scanning mode over a range of 30°–80°, 2 θ angel. The transmission electron microscopy (TEM) technique was used to observe *S. aureus* cells before and after exposure to silver nitrate solution. The cells were fixed and stained according to reported protocol (14). Then, imaging was done by two instruments of TEM (Philips CM120 TEM and Zeiss Leo 910 transmission electron microscope operating at 80 kV accelerating voltage and Gatan SC1000 camera). The size distribution and zeta potential of SNPs were analyzed by Dynamic Light Scattering (DLS) and Laser Doppler Velocimetry (LDV), respectively, using the Malvern Nano ZS instrument and the DTS software (Malvern Instruments, UK). The results are presented as mean. Each mean represents the average value of three measurements. Fourier transform infrared (FT-IR) spectrum of SNPs was recorded by Perkin Elmer Spectrum Two spectrometer using the KBr pellet technique. 

*Concentration determination of SNPs*

The solution concentration of SNPs was determined by table of extinction coeﬃcient, data of size, and optical spectrum according to the referred reference (18).

*In-vitro cytotoxicity assay*

Toxicity effect of SNPs was evaluated on human breast cancer cells (MCF-7) using the MTT assay. MCF-7 cells (1 × 10^4^ cells per well) were seeded onto a 96-well plate with RPMI 1640 medium supplemented with 10% fetal bovine serum, 100 units/mL penicillin, and 100 µg/mL streptomycin, incubated at 37 °C under a 5% CO_2_/95% air atmosphere for 24 h. Next, the medium of the cells was replaced with a fresh medium containing SNP in different concentrations (10–100 µg/mL) and the plates were incubated in 95% humidity, 5% CO_2_ at 37 °C for 24 h. Cytotoxicity was measured using 3-(4,5-dimethylthiazol-2-yl)-2,5 diphenyl tetrazolium bromide (MTT). Ten µL of MTT solution was added to each well and the plates were incubated for two hours in dark conditions, then 100 µL DMSO was added to solubilize the MTT. The absorbance of each well was measured at 570 nm with a microplate spectrophotometer [BioTek (Germany)]. The half maximal inhibitory concentration (IC_50_) is a measure of the potency of a substance in inhibiting a specific biological or biochemical function. Here, IC_50_ value is a concentration of SNPs (drug) that showed 50% reductions in cell viability and the relative viability was estimated by % of treated cells against untreated cells (as control group) (19). 

*In-vitro evaluation of the antimicrobial activity using agar diffusion assay*

Well diffusion method was used to observe antibacterial activity of SNPs on bacterial species (*S. aureus* strains (ATCC 6538p, 29737, and 25923), *Staphylococcus epidermidis* (*S. epidermidis*) (ATCC 12228), and *Escherichia coli* (*E. coli*) (ATCC 8739)). The bacterial strains were prepared by growing a single colony in LB medium overnight and adjusting the turbidity to 0.5 McFarland standard. About 100 μL from 0.5 McFarland standard was spread uniformly on Mueller-Hinton agar (MHA) plates and various concentrations of SNPs (50–400 µg/mL) were loaded into the wells with a diameter of 6 mm. These plates were incubated at 37 °C for 24 h. Zone of inhibition was determined by measuring the diameter of bacterial clearance after 24 h (20). The results are presented as mean of three measurements.

*Investigation of antibacterial activity using minimum inhibitory concentration (MIC)*

The MIC is the lowest concentration of antimicrobial agent that completely inhibits growth of the microorganism. MIC experiment was performed against the *S. aureus* (ATCC 6538p, 29737, and 25923) and *E. coli* (ATCC 8739) in different concentrations of SNPs (10, 30, 50, 70, 90, 110, 130, and 150 µg/mL). SNP solutions were added to wells of a 96-well plate already containing 100 µL of MH broth and 0.5 McFarland standard (~1 ×10^8 ^CFU·mL^−1^) of each bacterium. The wells included culture medium plus bacteria (for growth) and the control wells containing culture medium alone. MIC was determined after 24 h of incubation at 37 °C (20). 

The test was repeated three times and the results are presented as mean of three measurements. The MIC was considered minimum concentration of the nanoparticle that showed zero turbidity.

*Evaluation of SNP stability against aggregation* *reaction*

Sodium chloride solution (1 M) was added dropwise to the nanoparticle solution. Change of nanoparticle color was investigated and observations were recorded (21-22).

*Stability evaluation at various pHs*

Similar concentrations of the nanoparticles were prepared and UV-Vis data of nanoparticles were recorded. Next, pH of the solutions were adjusted over a wide range (4-7, 14) and the solutions were incubated for 24 h at room temperature. Next, UV-Vis spectrums were recorded (23).

*Statistical analysis*

Statistical calculations were performed using t-test. *P* < 0.05 was considered statistically significant.

## Results

*Characterization and purification of SNPs*

**Figure 1:**
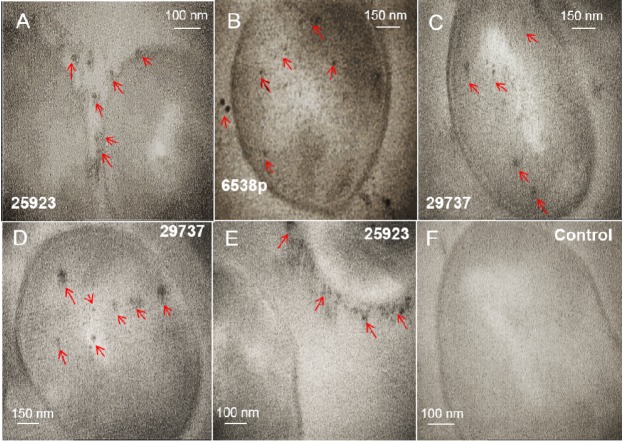
Intracellular biosynthesis of silver nanoparticles in cells of *Staphylococcus aureus *(ATCC 29737, 25923, and 6538p) is analyzed by TEM (A-E). The cells are not treated with silver nitrate (F). Separated SNPs from the cell walls was specified by arrows. These SNPs probably separated from the cell wall during preparation steps of the cells for imaging by TEM

**Figure 2:**
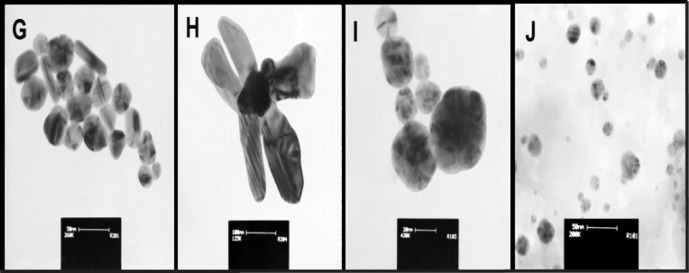
Morphology and size distribution of synthetic (G-H) and biosynthetic (I-J) silver nanoparticles (after extraction process) determined by TEM images. Scale bars are 50, 100, 20, and 50 nm respectively

TEM images from intracellular technique (Figure 1 and supplementary file, Figure S1) and extracellular technique (supplementary file, Figure S2) of biosynthesis using *S. aureus *were presented. In intracellular biosynthesis, the images displayed the presence of SNPs on outside surface of the cell walls and in the cytoplasm. *S. aureus* ATCC 29737 exhibited more accumulation of biosynthesized nanoparticles in the cell cytoplasm but strain of ATCC 25923 displayed more accumulation of the nanoparticles on outside surface of the cell walls (Figure 1 and supplementary file, Figure S1). TEM images of the supernatant mixture and AgNO_3 _in extracellular biosynthesis showed no SNPs (supplementary file, Figure S2). The images of purified SNPs were recorded after the recovery process (Figure 2). Growth behavior of the bacteria after the biosynthesis process was evaluated. All strains showed growth after the biosynthesis process (supplementary file, Figure S3).

**Figure 3:**
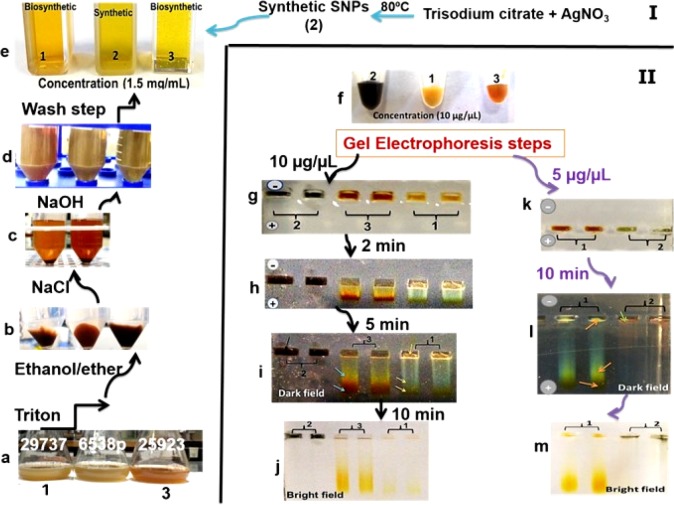
Designed procedure from intracellular biosynthesis to purification of silver nanoparticles (SNPs) (panel I). True color photograph, without staining. Samples 1 and 3 include biosynthetic SNPs. Sample 2 includes synthetic SNPs. Bacteria cultures containing biosynthetic SNPs (panel I (a)). Purification procedure (panel I (b-d)). Concentrated solutions of synthetic and biosynthetic SNPs (panel II (f)). Gel electrophoresis steps (panel II). The gels show different colors in SNP lanes and clear separation due to different sizes of SNPs (panel II (h-i, l))

All steps of the recovery process of intracellular SNPs were presented in Figure 3 (panel I, II). In Figure 3, panel (I) exhibits the recovery steps and panel (II) exhibits gel electrophoresis steps. The images of purified biosynthetic nanoparticles confirmed the formation of spherical nanoparticles with size distribution ranging between 5 to 40 nm (ATCC 25923 and 6538p), and 10–50 nm (ATCC 29737) but synthetic SNPs exhibited size distribution between 10 and 100 nm with various shapes (spherical, triangular, hexagonal, rod, oval and flower shape) (Figure 2). The DLS data confirmed size distribution of biosynthetic SNPs. UV spectrum related to the supernatant of the purification step (ethanol:diethyl ether) indicated absorption of lipid below the 300 nm range (15). Color of synthetic nanoparticles at low concentration (1.5 µg/mL) (Figure 3e) was yellow, while it became black at high concentration (10 µg/mL) (Figure 3f). The images of gel electrophoresis were presented at both low and high concentrations (Figure 3II). The biosynthetic SNPs moved quickly whereas synthetic SNPs retarded. Synthetic SNPs did not move even 10 min after running at 10 µg/mL (Figure 3II). Gel showed separation into different colors. Synthetic SNPs displayed no band at 10 µg/mL, but indicated one maroon band at 5 µg/mL (Figure 3l), which became black in the presence of bright light (Figure 3m). Biosynthetic SNPs indicated different colors in the dark field (in the presence of low light) and one yellow band in their bright field (in the presence of bright light), which was stable for 2 days against bright light. The position of horizontal colors on the gel was specified by arrows (Figures 3i and 3l). Zeta potential of the SNPs was -30 ± 3 and -35 ± 2 mV for synthetic and biosynthetic SNPs, respectively. 

**Figure 4:**
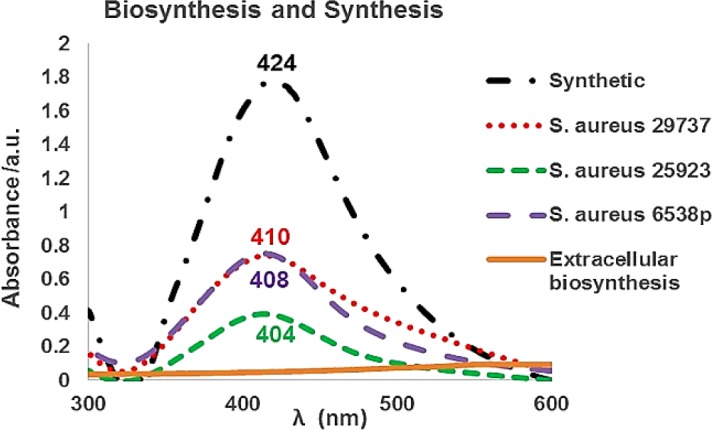
UV-Vis spectra of synthetic silver nanoparticles (SNPs) and biosynthetic SNPs using *Staphylococcus aureus *(*S. aureus*) intracellularly. In extracellular biosynthesis, culture flasks were incubated under either with or without bright light. The obtained supernatants did not show any absorption in the presence of AgNO3 at 350–600 nm range. Also, the obtained supernatants of KNO3- treated culture ﬂasks revealed no absorption in the presence of AgNO3 at 350–600 nm range

After purification process, SNPs were analyzed by a UV-Vis spectrometer. Biosynthetic nanoparticles showed SPR peaks at 404 to 410 nm range. Prepared SNPs by (synthetic SNPs) exhibited SPR peak at 424 nm (Figure 4). The UV-Vis spectrum of AgNO_3_-treated supernatant of the extracellular biosynthesis method showed no absorption at 350–600 nm. In extracellular biosynthesis, the flasks were incubated in the presence of bright light and dark condition separately. The supernatants containing AgNO_3_ did not show any absorption at 350–600 nm range (Figure 4), and the presence or absence of bright light had no effect on the biosynthesis process of SNPs. Also, the treated culture ﬂasks with 10 mmol KNO_3_ and AgNO_3_ revealed no absorption at 350–600 nm.

X-ray diffraction of biosynthetic nanoparticles was carried out (supplementary file, Figure S4). A comparison of XRD spectrum with the previous reports conﬁrmed that SNPs had nanocrystal form, as evidenced by the correspondence of peaks at values of 39.01°, 46.48°, 64.69°, and 77.62° to [111], [200], [220], and [311], respectively, for silver (JCPDS file no. 04–0783).

In order to characterize surface material of nanoparticles (stabilizer material), FT-IR spectrum of SNPs was prepared (supplementary file, Figure S5). *S. aureus* ATCC 6538p and 25923 exhibited the same pattern in IR spectrum. As shown, FTIR spectra indicated main biomolecules. The C-O absorption of glycogen and other carbohydrates, phospholipids, and nucleic acid groups mainly occur at the 1250–1000 cm^-1 ^wavelength range. The absorption of CH_2_ and CH aliphatic groups and the lipid acyl chains have appeared at 3050–2800 cm^-1^ and 1500–1350 cm^-1^ and around 1740 cm^-1^ for the ester carbonyl absorption (24). Moreover, amide I vibration near 1650 cm^−1 ^and amide II bands around 1557 nm were revealed. These absorptions were attributed to the C=O stretching structure, while the NH stretching vibration gave absorption between 3070–3300 cm^-1^. The wide band in the range of 3000–3400 cm^−1^ appeared from the -NH_2_ and -OH groups in protein molecules (25). 

*Antibacterial and cytotoxic activity of the SNPs*

**Figure 5:**
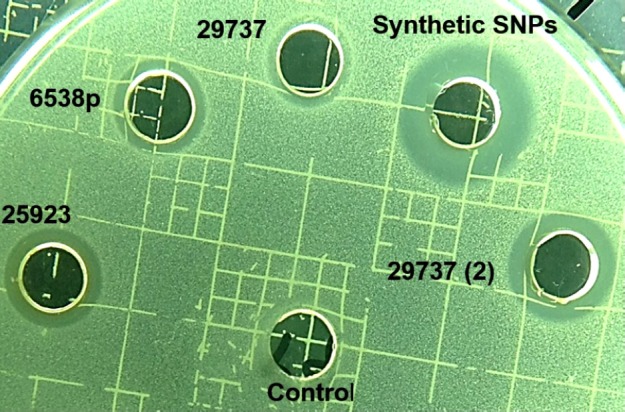
Image of agar diffusion test. *Staphylococcus aureus *ATCC 29737 was added to Mueller-Hinton agar medium. 200 µg/mL of Synthetic and biosynthetic silver nanoparticles was added to the wells. (2) 400 µg/mL of the biosynthetic SNPs was added to the well

**Figure 6:**
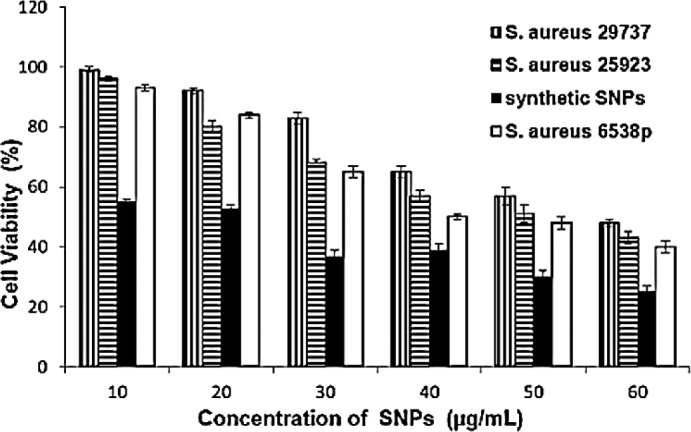
Cytotoxicity assay of MCF-7 cells with various concentrations of synthetic and biosynthetic silver nanoparticles in a 96-well plate and 24 h incubation at 37 °C. Cell viability data are presented as the mean ± SD of triplicates. Multiple measurement comparisons using Student’s t-test are considered statistically signiﬁcant when *P *< 0.05

To determine antibacterial activity of SNPs, MIC, and agar diffusion tests were used (Table 1 and Figure 5). Synthetic nanoparticles showed remarkable antibacterial activity in agar diffusion assay at various concentrations (50–200 µg/mL), while biosynthetic SNPs exhibited weak antibacterial activity (only at 200 µg/mL of SNPs) against tested strains. Inhibition zones of biosynthetic SNPs were between 10 and 14 mm, while inhibition zones of synthetic ones were between 16 and 19 mm (Table 1 and Figure 5). Data of MIC assay confirmed agar diffusion results. MIC values of biosynthetic SNPs against bacteria were 105 ± 2 (for *S. aureus*) and 120 ± 2 µg/mL (for *E. coli*), while for synthetic nanoparticles indicated 35 ± 2 µg/mL for *E.* *coli* and 30 ± 2 for *S. aureus*. In addition, the cytotoxicity of SNPs was tested against the MCF-7 cancerous cell line (Figure 6). Synthetic and biosynthetic nanoparticles showed cytotoxic effect against MCF-7 cell line. The half maximal inhibitory concentration (IC_50_ value) was 20 ± 3 µg/mL for synthetic SNPs, but this value was between 50 and 60 µg/mL for biosynthetic nanoparticles against the tested strains.

**Table 1:** Antibacterial activity of synthetic and biosynthetic silver nanoparticles was investigated by agar diffusion assay (inhibition zone diameter) against *Staphylococcus aureus* (ATCC 6538p, 29737, and 25923), *Staphylococcus epidermidis *(ATCC 12228), and *Escherichia coli* (ATCC 8739)

a **ATCC**	**Well content (µg/mL)**	**Inhibition zone diameter (mm ± SD)**				
		*S. aureus*		*E. coli*		*S. epidermidis*
		29737	25923	6538p		
a6538p	200	12 ± 1	-	12 ± 1	12 ± 1	14 ± 1
a29737	200	10 ± 1	-	-	10 ± 1	12 ± 1
a25923	200	14 ± 1	11 ± 1	-	12 ± 1	13 ± 1
bChemical	200	19 ± 2	19 ± 1	18 ± 2	16 ± 1	19 ± 1

a *S. aureus *strain in biosynthesis of silver nanoparticles*.*

b Synthetic silver *nanoparticles. *(-) The strain was not tested.

*Evaluation of the SNPs stability*

NaCl solution (1 M) was added to the nanoparticle solution in DDW. The addition of 10 µL of NaCl solution caused color changes from yellow to gray and black in the synthetic nanoparticles solution, and no color change of the biosynthetic SNPs were observed even at higher concentration (500 µL of NaCl solution). The visible absorption spectrum of synthetic nanoparticles after addition of 10 µL of NaCl solution showed a weak peak at 370–600 nm, but the visible absorption spectrum of biosynthetic nanoparticles showed absorption peaks at 404–414 nm, even at higher NaCl concentration (data are not shown). pH of solutions was adjusted over a wide range (4-7, 14). The solutions were incubated for 24 h at room temperature and then the UV-Vis spectra were recorded. UV-Vis spectrum of alkaline solution (pH 14) of synthetic SNPs against biosynthetic SNPs appeared more sensitive, but at 3–5 pH range both synthetic and biosynthetic SNPs were sensitive and showed reduction at absorption peaks at 350-600 nm (supplementary file, Figure S6). 

## Discussion

*S.* *aureus* (ATCC 6538p, 29737 and 25923) was evaluated for its ability to biosynthesize SNPs intra- and extracellularly. Effect of nitrate ion and light on the process of extracellular biosynthesis was assessed. A simple method was employed to purify biosynthetic nanoparticles in the intracellular technique. Characterization of SNPs was determined by TEM, XRD, UV-Vis, and FT-IR methods. Biological and non-biological properties of synthetic and biosynthetic nanoparticles were compared together to determine their potential applications.

The intracellular mechanism of nanoparticle biosynthesis by microorganisms is not clearly understood. It was reported that the first step involves trapping of ions on the cell surface by electrostatic interaction between ions and charged groups in enzymes of the cell (26). Sugars and enzymes as reducing agents at the cell wall cause reduction of metal ions to nanoparticles (14, 26). Nanoparticles were protected by a layer from charged functional groups of the cell wall. Some ions and small nanoparticles could diffuse across the cell wall, localizing on the cytoplasmic membrane. Enzymes that are present in the cytoplasm reduce metal ions (26). Crystal growth occurs inside of the cell, hence, larger nanoparticles formed within the cell (1). The nitrate reductase enzyme was known as an effective enzyme in biosynthesis of SNPs. Nitrate reductase was employed to shuttle electron from nitrate to the metal ion (1, 14 and 27-28). Biochemical tests indicate *S. aureus* wild type carries out a positive reaction to the nitrate reductase test (29). TEM images (Figure 1 and supplementary file, Figure S1) of treated microorganisms before and after the addition of the silver nitrate solution, confirmed intracellular formation of SNPs. *S. aureus* strains tend to form small and uniform SNPs. Among strains tested, *S. aureus* 25923 biosynthesized SNPs more on outside of the cell walls, while strain 29737 exhibited SNPs more in the cytoplasm. TEM images (Figure 1) displayed some SNPs in extracellular space. These SNPs were probably separated from the cell wall during preparation steps of the cells for imaging by TEM. 

The mechanism of extracellular synthesis of nanoparticles using microorganisms is basically found to be a nitrate reductase-mediated synthesis. This enzyme is present in the cell-free supernatant of cultures and helps in the bioreduction of metal ions and synthesis of nanoparticles (3, 6 and 28). Addition of nitrate ion to the culture medium caused more nitrate reductase enzyme activity in the *Propionibacterium *culture (30). Biosynthesis of SNPs using *E. coli* was least in LB broth medium and higher in nitrate broth (31). Further studies confirmed an NADH-dependent reductase was associated with reduction of Ag^+^ to Ag^0^ in the case of fungi (16). Although supernatant can contain the enzyme nitrate reductase, it is less likely that NADPH is present in the supernatant (1). In extracellular synthesis of gold nanoparticles using *Rhodopseudomonas capsulate,* a similar mechanism was reported. The bacterium *R. capsulata* was known to secrete cofactor NADH and NADH-dependent enzymes. The reduction of gold ions was initiated by the electron transfer from the NADH by NADH-dependent reductase as electron carrier. Then the gold ions obtain electrons and are reduced to Au^0^ (6). In this study, in the extracellular biosynthesis method, no SNPs was formed. None of the strains could produce the nanoparticles under all conditions (dark, bright light, and presence of nitrate ion) (Figure 4 and supplementary file, Figure S2). The results of UV-Vis spectra showed that addition of the nitrate ion into culture medium did not improve the formation of the nanoparticles in extracellular biosynthesis (Fig. 4). It is possible that enzyme component, or enzyme system is not functioning extracellularly or were not probably present in cell-free supernatant of *S. aureus* (ATCC 6538p, 29737, and 25923).

SNPs have low stability and are sensitive to aggregation (21-23, 32) and oxidation reaction (33). Coating of SNPs by a proper protective layer can effectively stabilize SNPs. This layer stabilizes nanoparticle solutions to exist at high NaCl concentrations (34) and over a wide pH range (32). A designed peptide and a thioalkylated poly (ethylene glycol) were used to stabilize SNPs in water. The particle aggregation reactions were prevented and nanoparticle solutions were stable in the presence of high concentrations of NaCl and over a wide pH range (35). Here, experimental methods related to stability of SNPs were performed according to our previous study (15). The synthetic and biosynthetic nanoparticles exhibited different stability in the presence of NaCl solutions. Synthetic SNPs aggregated at low concentration of NaCl (0.1 mM), but biosynthetic ones showed high stability even in the presence of NaCl solution (5 mM). At alkaline pH range, biosynthetic nanoparticles were stable for more than 24 h (supplementary file, Figure S6). The synthetic SNPs retarded more than the biosynthetic SNPs in gel electrophoresis. The biosynthetic and synthetic SNPs were not different in zeta potential significantly (-30 ± 3 and -35 ± 2 mV for synthetic and biosynthetic SNPs, respectively), but synthetic SNPs did not move even after 10 min of electrophoresis at 10 µg/mL concentration (Figure 3II). Gel showed a separation into different colors. Synthetic SNPs displayed one maroon band (at 5 µg/mL) (Figure 3l), but biosynthetic SNPs indicated two bands in low light (dark field) (Figures 5i and 5l). The colors are due to the size-dependent optical properties of SNPs (15). Synthetic nanoparticles at high concentration and inside the gels were black (Figures 3f, 3g-3j). The inability of synthetic nanoparticles to move on the gel and their color can be due to aggregation reaction. In the synthetic method, the nanoparticles were synthesized by the chemical reduction of AgNO_3_ using NaBH_4_. The borohydride anions were adsorbed onto the small particles (12, 14). Data of FT-IR spectra (supplementary file, Figure S5) of biosynthetic nanoparticles indicated biomolecules exist on the surface of nanoparticles. Biomolecules are polymeric, biocompatible, and non-toxic in nature. The results of this study showed that biomolecules present on the surface of biosynthetic SNPs could cause more stability than borohydride anions on the surface of synthetic SNPs. 

SNPs are efficient at absorbing and scattering of light. The optical and electronic properties of nanocrystals are dependent on physical properties such as nanoparticle diameter, size distribution, shape, and crystallinity. Control of these properties is a challenge in the methods of nanoparticle synthesis and biosynthesis (11). The experimentally measured spectra are dependent on nanoparticle shape of silver. Shape-controlled nanoparticles enabled new plasmonic and sensing applications (36). Although, many methods were reported to prepare uniform shapes and small size distribution of SNPs but most methods are very sensitive to environmental and experimental conditions. So unintentional change of the conditions cause unwanted shape and size of SNPs. For example, silver nitrate was reduced rapidly by NaBH_4_. Reaction conditions including stirring time and relative quantities of reagents must be carefully controlled in presence of NaBH_4 _(12). Unintentional change in concentration of NaBH_4_, reaction temperature, reaction time, and contaminated container caused the production of various shapes and diverse-size distribution (12-13). The performance of trisodium citrate as reducing agent is dependent on time, pH, temperature and concentration (15, 37-38). In extracellular biosynthesis, shape and size nanoparticles could be changed by changing pH or temperature of the reaction mixture (39). Often, extracellularly produced nanoparticles have size distribution between 10 nm and 6 µm with various shapes (spherical, triangular, hexagonal, and plate) (7-10, 39-40), while intracellularly produced nanoparticles have size distribution less than 50 nm with spherical shape (1, 7-10 and 15). Most methods of intracellular biosynthesis are performed at pH 7-8 and ambient temperature. Here, biosynthesized nanoparticles indicated size distribution between 5 and 50 nm with uniform shape (spherical) (Figures 1 and 2).

Nanoparticles attach and penetrate into the cell wall and damage it (40-42). Various theories have been reported for actions of SNPs on microbes. SNPs cause structural changes in the cell membrane (43-45). *In-vitro* cytotoxicity assay of biosynthetic SNPs was investigated on the MCF-7 cell line by MTT assay. SNPs were synthesized using extracts of *Sesbania grandiﬂora *(46) and* Achillea biebersteinii* extracellularly (19) and the inhibitory concentration (IC_50_ value) was obtained at 20 µg/mL after 24 h of cell treatment with SNPs (19, 46). There was an immediate induction of cellular damage in terms of loss of cell membrane integrity, oxidative stress, and apoptosis in the cells treated with SNPs (46). Also, SNPs were synthesized using *Annona squamosa* extract, which were reportedly cytotoxic against MCF-7 cells (IC_50_ 50 µg/mL after 24 h) (47). The FT-IR spectra showed that proteins, phenolic compounds (19, 47), and biocompounds (46) were present on the surface of SNPs and protected the SNPs from aggregation, and thereby retained the long stability of nanoparticles. Here, IC_50_ was 20 ± 3 µg/mL for synthetic SNPs and between 50 and 60 µg/mL for intracellular biosynthetic SNPs (Figure 6). Extracellularly prepared SNPs (using *Pilimelia columellifera subsp*) exhibited MIC of 40 µg/mL against *E. coli *and 70 µg/mL against *S. aureus* (48), while we obtained MIC values between 105 ± 3 (for *S. aureus*) and 120 ± 3 µg/mL (for *E. coli*) using biosynthetic nanoparticles. Synthetic nanoparticles exhibited lower MIC values, 35 ± 2 µg/mL against *E. coli* and 30 ± 2 µg/mL against *S. aureus*.

*S. aureus* (ATCC 6538p, 29737, and 25923) only biosynthesized SNPs intracellularly. *S. aureus* tended to form smaller and uniform SNPs (spherical) than synthetic SNPs. In the extracellular biosynthesis, none of the strains could produce the nanoparticles under all conditions tested (dark, bright light, and presence of nitrate ion). It was possible that enzyme component, or enzyme system were not functioning extracellularly or were not probably present in the cell-free supernatants of *S.* *aureus* (ATCC 6538p, 29737, and 25923). The result demonstrate that the intracellular method of biosynthesis is more efficient in producing spherical SNP with small-size distribution and can be efficient for the reduction of SNP toxicity and the increase of its stability. These nanoparticles may be useful for being employed as biosensors. 
