# Supplementary material for: Does Biosynthetic Silver Nanoparticles Are More Stable With Lower Toxicity than Their Synthetic Counterparts?
Source: Iran J Pharm Res. 2019 Winter;18(1):210–21. (PMC6487435)
Supplement: Supplementary file 1 — supplementary material [file ijpr-18-210-s001.pdf]

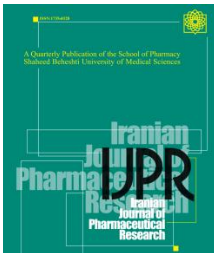

Supplementary Materials for

Does biosynthetic silver nanoparticles are more stable with lower toxicity than their synthetic counterparts?

Zohreh Rezvani Amin, Zahra Khashyarmanesh, Bibi Sedigheh Fazly Bazzaz* and Zahra Sabeti Noghabi

*To whom correspondence should be addressed. E-mail: fazlis@mums.ac.ir

Volume 18, Issue 1 (Winter 2019)

This PDF file includes:

Experimental

Results: Figures S1 to S6.

Experimental

Biosynthesis of SNPs using S. aureus (intracellularly) and purification steps

The cultures of *S. aureus* strains (ATCC 6538p, 29737, and 25923) were used for biosynthesizing SNPs. A full loop of each bacteria was grown in the 40 mL Luria-Bertani (LB) broth medium (US, Thermo Fisher Scientific) at pH 7.5, 35 °C, and 150 rpm in shaker incubator for 24 h (1). Then 100 mL fresh LB broth and 10 mL aqueous solution of silver nitrate (2 mM) were added to each culture. pH of the cultures were adjusted at 8 and the cultures were grown at 35 °C and 150 rpm for a further 24 h. After color change of the cultures (from white to brown and dark brown); they were incubated at room temperature for further 4 h. The contents of each flask was centrifuged (15 min at 3634×g). Prepared pellet was washed with phosphate buffered saline (PBS) (1X) and was lysed by 5 mL of Triton-X100 (2%) solution (on ice for 30–35 min with frequent mixing of the components by a mixer). Centrifugation (3634×g for 15 min) was used to remove the triton solution, the precipitate was washed by DDW and the supernatant was eliminated (3634×g for 15 min). The mixture of ethanol:diethyl ether (3:1) (10 mL) was added to the rest, stirred for 5 min, and the supernatant was removed (3634×g for 10 min). NaCl (2 M, 5 mL) was added to the precipitate, stirred for 1 min, and the supernatant was removed (5232×g for 10 min). The solution of NaOH (2 M, 10 mL) was added to the precipitate, stirred for 5 min, and stored in hot water (60–70 °C) with frequent mixing of the components by a mixer. As soon as the opaque solution became fully transparent, the solution was centrifuged at 9302×g for 15 min, the supernatant wasdiscarded, 40 mL of DDW was quickly added to the rest, and stirred for 3 min. Next, the supernatant was discarded (9302×g for 15 min) and this step was repeated 2 more times. DDW (15 mL) was added to biosynthetic SNPs and the final solutions were stored at 5 °C and in dark conditions (2).

Gel electrophoresis was employed for characterization of SNPs (3). Gel electrophoresis separated SNPs by a 0.7% agarose gel (15 cm electrode spacing, ran for 10 min at 150 V) in Tris/borate/EDTA (TBE) buffer (0.5 X) at pH 9. Two concentrations of SNPs (about 7 µL from 10 and 5 µg/µL) were added to the gel wells separately. The steps were performed in weak light conditions.

Growth behavior of the bacteria after the biosynthesis process

To investigate the growth behavior of each bacterium after SNP biosynthesis, a loop full of the final step (intracellular biosynthesis) which produced biosynthetic nanoparticles was streaked on MHA plates. This was performed for all bacteria separately. The plates were incubated at 35 °C for 24 h.

Concentration measurement of SNPs

The solution concentration of SNPs was determined by table of extinction coefficient, data of size, and optical spectrum according to referred reference (4).

Results

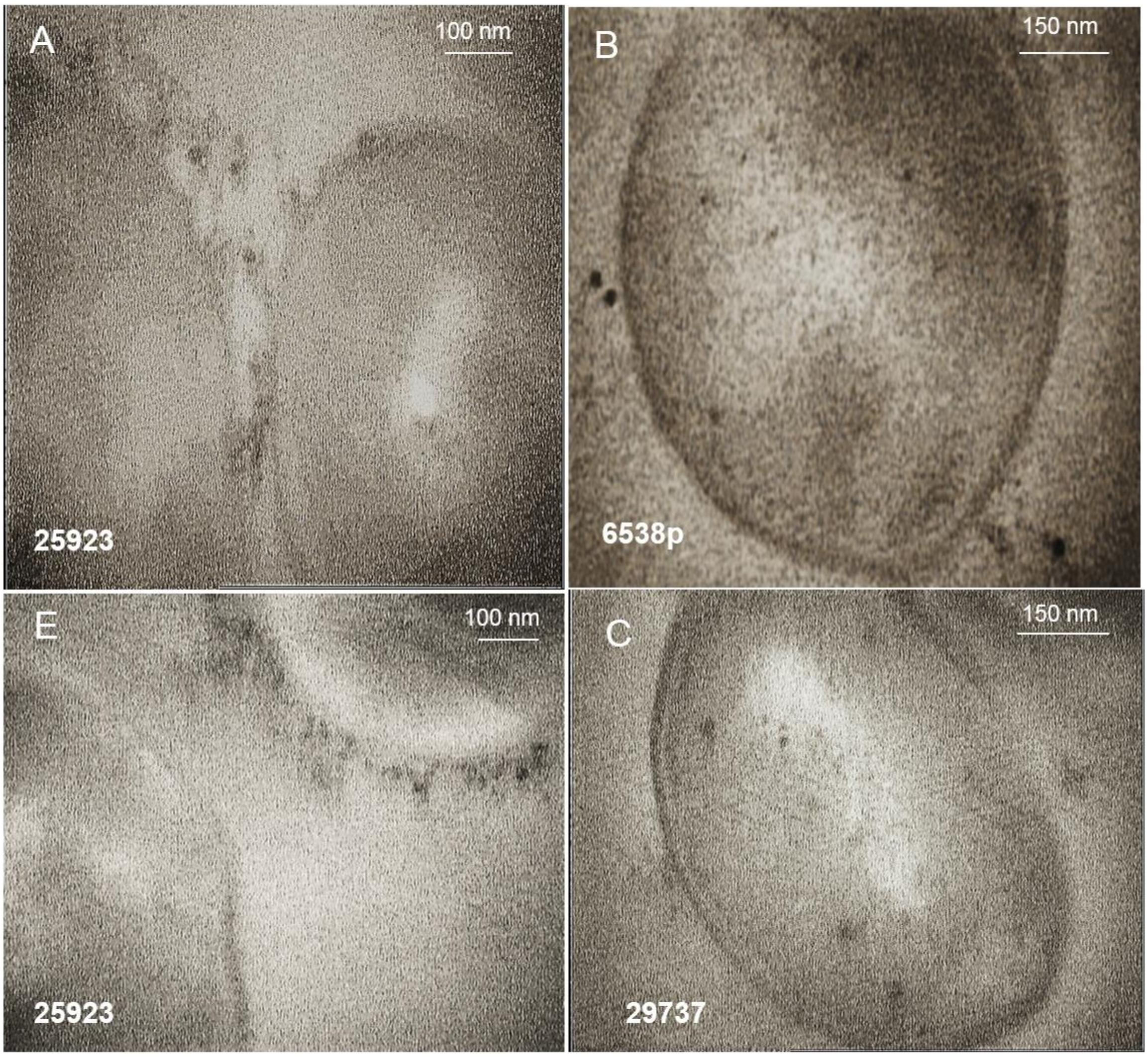

Figure S1. Intracellular biosynthesis of silver nanoparticles in cells of *Staphylococcus aureus* (ATCC 29737, 25923, and 6538p) is analyzed by TEM. Probably separated SNPs from the cell walls separated from the cell wall during preparation steps of the cells for imaging by TEM.

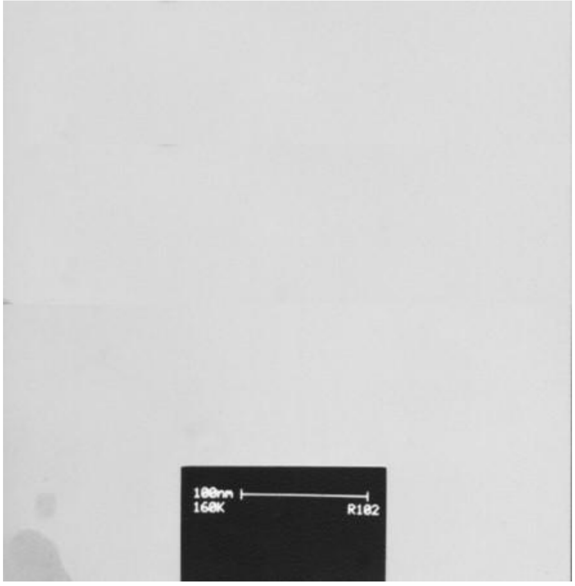

Figure S2. TEM image of the mixture of silver nitrate and the supernatant after 24 h incubation. In extracellular biosynthesis, culture flasks were incubated under either with or without bright light. The obtained supernatants did not show any silver nanoparticle (SNP) in the presence of $AgNO_3$. Also, the obtained supernatants of KNO_3-treated culture flasks revealed no SNP in the presence of $AgNO_3$ in TEM images.

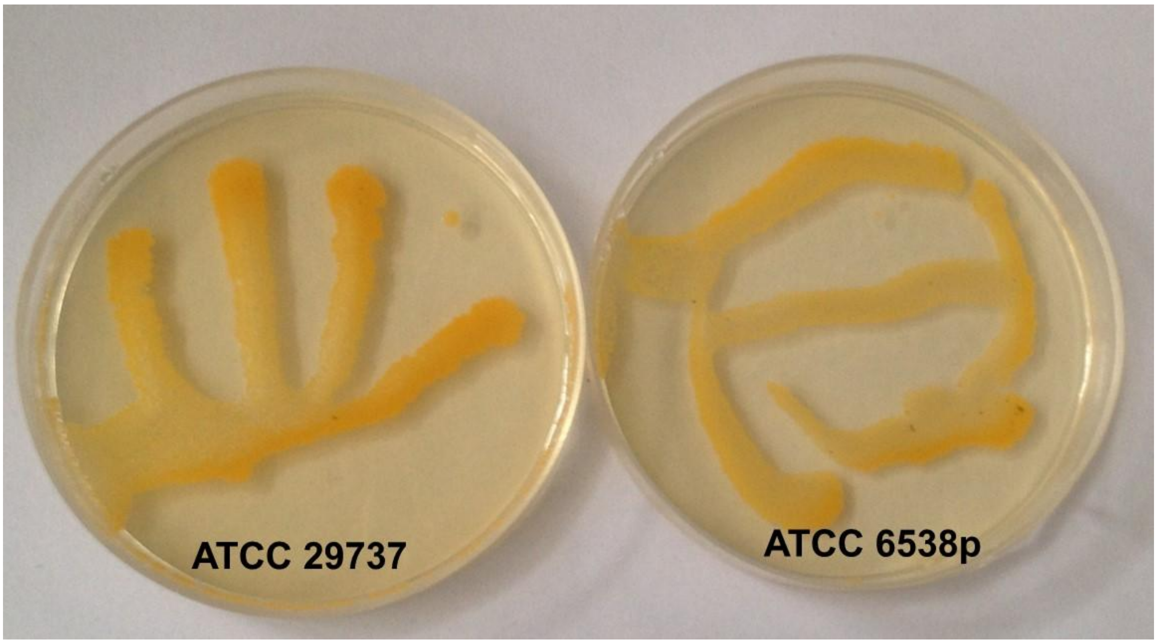

Figure S3. Growth behavior of the bacteria (*Staphylococcus aureus*) was investigated after biosynthesis process on Mueller Hinton agar (MHA) plates (all data are not shown).

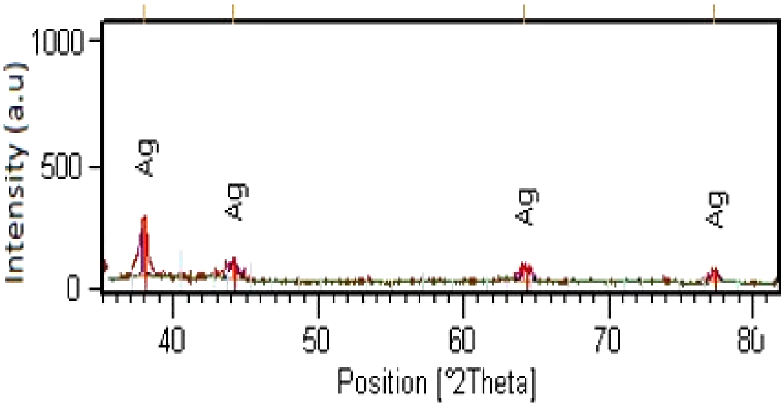

Figure S4. X-ray diffraction of biosynthesized silver nanoparticles (SNPs) using *Staphylococcus aureus* (ATCC 25923) intracellularly. X-ray diffraction data of biosynthetic SNPs by *S. aureus* strains (ATCC 25923, 29737, and 6538p) are alike.

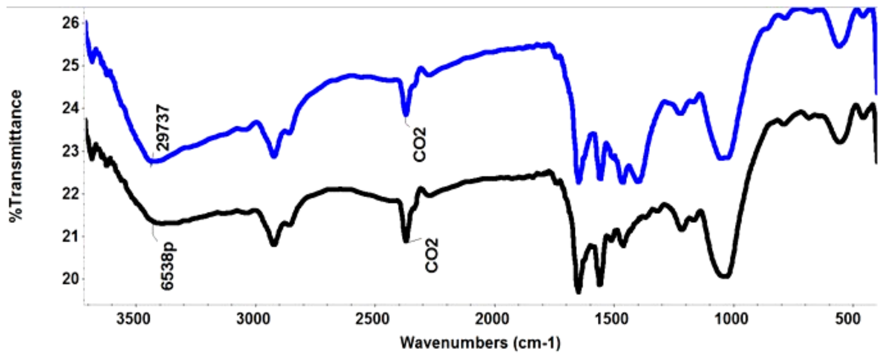

Figure S5. FT-IR spectra related to biosynthesized nanoparticles using *Staphylococcus aureus* intracellularly. 25923 and 6538p strains show similar spectra.

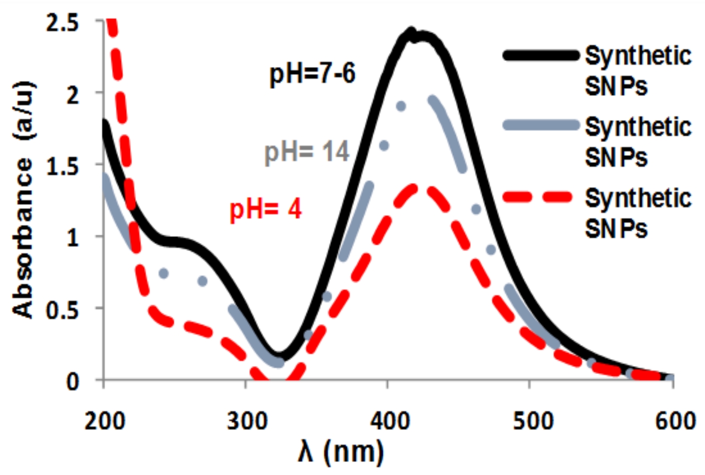

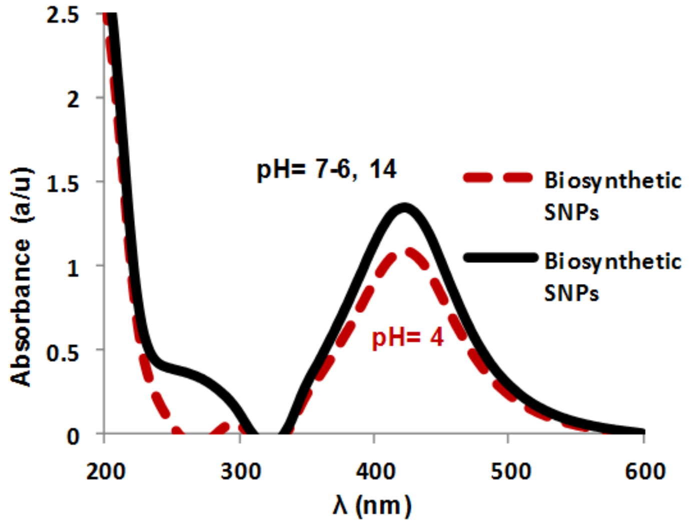

Figure S6. UV-VIS Spectra of synthetic and biosynthetic silver nanoparticles (SNPs) at various pHs. The samples were incubated 24 h at various pHs, then the spectra were recorded.
